# Evolution of *Trypanosoma cruzi*: clarifying hybridisations, mitochondrial introgressions and phylogenetic relationships between major lineages

**DOI:** 10.1590/0074-02760140401

**Published:** 2015-05

**Authors:** Nicolás Tomasini, Patricio Diosque

**Affiliations:** Instituto de Patología Experimental, Universidad Nacional de Salta, Salta, Argentina

**Keywords:** Chagas disease, parasite, phylogeny, Trypanosomatidae, discrete typing units

## Abstract

Several different models of Trypanosoma cruzi evolution have been proposed. These models suggest that scarce events of genetic exchange occurred during the evolutionary history of this parasite. In addition, the debate has focused on the existence of one or two hybridisation events during the evolution of T. cruzi lineages. Here, we reviewed the literature and analysed available sequence data to clarify the phylogenetic relationships among these different lineages. We observed that TcI, TcIII and TcIV form a monophyletic group and that TcIII and TcIV are not, as previously suggested, TcI-TcII hybrids. Particularly, TcI and TcIII are sister groups that diverged around the same time that a widely distributed TcIV split into two clades (TcIV_S_ and TcIV_N_). In addition, we collected evidence that TcIII received TcIV_S _kDNA by introgression on several occasions. Different demographic hypotheses (surfing and asymmetrical introgression) may explain the origin and expansion of the TcIII group. Considering these hypotheses, genetic exchange should have been relatively frequent between TcIII and TcIV_S_ in the geographic area in which their distributions overlapped. In addition, our results support the hypothesis that two independent hybridisation events gave rise to TcV and TcVI. Consequently, TcIV_S_ kDNA was first transferred to TcIII and later to TcV and TcVI in TcII/TcIII hybridisation events.

*Trypanosoma cruzi*, the etiological agent of Chagas disease, affects several million people around the world. The major phylogenetic subdivisions of *T. cruzi* were widely analysed by Miles et al. (1977, 1978), who described different zymodemes by multilocus enzyme electrophoresis (MLEE). A few years ago, six different discrete typing units (DTUs) were clearly defined for *T. cruzi* based on different genetic markers (Zingales et al. 2012). These DTUs were termed from TcI to TcVI (Zingales et al. 2009). Recently, an additional DTU that is mainly associated with bats was proposed and named TcBat (Marcili et al. 2009a). The relationships between these DTUs were explained by several models, but these models are contradictory on several points (Barnabe et al. 2000, Westenberger et al. 2005, de Freitas et al. 2006, Flores-Lopez & Machado 2011, Lewis et al. 2011). Consequently, the origins of different DTUs and their inter-relationships remain controversial. In this paper, we analysed our own DNA sequence data of *T. cruzi* and data published by others to clarify the relationships between different DTUs. In addition, we discuss different evolutionary scenarios for *T. cruzi* and propose a model for the origin of each DTU.

## MATERIALS AND METHODS

*Analysed sequences* - In a previous paper about multilocus sequence typing (MLST) for *T. cruzi*, we analysed 13 housekeeping gene fragments by simple neighbour-joining (NJ) analysis with the goal of obtaining a standardised MLST method for DTU assignment (Diosque et al. 2014). These sequences were reanalysed in the current work. The GenBank accessions are as follows: JN129501-JN129502, JN129511-JN129518, JN129523-JN129524, JN129534-JN129535, JN129544-JN129551, JN129556-JN129557, JN129567-JN129568, JN129577-JN129584, JN129589-JN129590, JN129600-JN129601, JN129610-JN129617, JN129622-JN129623, JN129633-JN129634, JN129643- JN129650, JN129655-JN129656, JN129666-JN129667, JN129676-JN129683, JN129688-JN129689, JN129699-JN129700, JN129709-JN129716, JN129721-JN129722, JN129732-JN129733, JN129742-JN129749, JN129754-JN129755, JN129765-JN129766, JN129775-JN129782, JN129787-JN129788, JN129798-JN129799, JN129808-JN129815, JN129820-JN129821 and KF889442-KF889646. Additionally, we used *T. cruzi marinkellei* as an outgroup. Sequence data of the selected targets for *T. cruzi marinkellei* were obtained from TriTrypDB (available from: tritrypdb.org) under the following accessions: TcMARK_CONTIG_2686, TcMARK_CONTIG_670, TcMARK_CONTIG_1404, Tc_MARK_2068, Tc_MARK_3409, Tc_MARK_5695, Tc_MARK_9874, Tc_MARK_515, Tc_MARK_4984, Tc_MARK_5926, Tc_MARK_8923, TcMARK_CONTIG_1818 and Tc_MARK_2666. In addition, sequences analysed by Westenberger et al. (2005) corresponding to *loci 1F8* calcium-binding protein, histone *H1*, histone *H3* and heat-shock protein 60 (*HSP60*) were downloaded from GenBank. The accessions for these sequences are the following: 1F8 (AF545071, AF545072, AF545074, AY540692, AY540693, AY540698, AY540699, AY540700, AY540703, AY540704, AY540705 and AY540706), *H1* (AF545075, AF545076, AF545077, AF545078, AY540672, AY540673, AY540675, AY540676, AY540677, AY540678, AY540679 and AY540680), *H3 *(AF545087, AF545088, AF545089, AF545090, AY540681, AY540682, AY540683, AY540684, AY540686, AY540687, AY540688, AY540689 and AY540690) and *HSP60 *(AY540716, AY540717, AY540718, AY540719, AY540720, AY540721, AY540722, AY540723, AY540724, AY540725, AY540726, AF545091, AF545092 and AF545093). Additionally, we analysed 97 cytochrome b (*CytB*) sequences published in Brisse et al. (2003) and Marcili et al. (2009b, c). The accessions are as follows: AJ130927, AJ130928, AJ130929, AJ130930, AJ130931, AJ130932, AJ130933, AJ130934, AJ130935, AJ130936, AJ130937, AJ130938, AJ439719, AJ439720, AJ439721, AJ439722, AJ439723, AJ439724, AJ439725, AJ439726, AJ439727, EU856367, EU856368, EU856369, EU856370, EU856371, EU856372, EU856373, EU856374, EU856374, EU856375, EU856376, EU856377, EU856378, EU856379, EU856380, FJ002253, FJ002254, FJ002255, FJ002256, FJ002257, FJ002258, FJ002259, FJ002260, FJ002261, FJ002262, FJ002263, FJ156759, FJ168768, FJ183398, FJ183399, FJ183400, FJ183401, FJ549386, FJ549387, FJ549388, FJ549389, FJ549390, FJ549391, FJ549392, FJ549393, FJ549394, FJ549395, FJ549396, FJ549397, FJ549398, FJ549399, FJ549400, FJ549401, FJ555631, FJ555631, FJ555632, FJ555633, FJ555633, FJ555634, FJ555635, FJ555636, FJ555637, FJ555638, FJ555639, FJ555640, FJ555641, FJ555642, FJ555643, FJ555644, FJ555645, FJ555646, FJ555647, FJ555648, FJ555649, FJ555650, FJ555651, FJ900246, FJ900247, FJ900248, JN543701 and JN543702. Finally, the cytochrome c oxidase subunit II**-**NADH dehydrogenase 1 (*COII-Nd1*) sequences analysed by Lewis et al. (2011) were as follows: HQ604870, AF359053, HQ604875, AF359032, HQ604873, AF359030, HQ604877, AF359046, AF359041, HQ604909, HQ604911 and HQ604907. For analyses requiring an outgroup, sequences from *T. cruzi marinkellei* strain TcMB7 were downloaded from Tritryp (available from: tritrypdb.org) database using a BLAST search strategy.

*Data analysis* - Alignments were produced with MEGA 6.0 software (Tamura et al. 2013) using default parameters. Regions with gaps in the alignment were excluded from the analyses. Concatenation of *CytB* and *COII-Nd1* fragments was made using MLSTest 1.0 (Tomasini et al. 2013). A five-nucleotide gap present in the sequences of three strains in the *COII-Nd1* alignment was coded as "G" for present and "A" for absent to be considered in the phylogenetic analysis. Sequences obtained in our previous paper (Diosque et al. 2014) were concatenated before performing most of the phylogenetic analyses. To evaluate congruence among different *loci* and suitability for concatenation, we performed a BioNJ-ILD test (Zelwer & Daubin 2004) with 1,000 random permutations. NJ analyses were made with MLSTest software using uncorrected p-distances and considering heterozygous sites as average states. One thousand bootstrap replications were used to evaluate branch support. Maximum likelihood (ML) analyses were conducted with MEGA 6.0 software. The best model for each analysis was selected using corrected Akaike information criterion implemented in jMODELTEST software (Posada 2008). Bayesian analyses were run in MrBayes v.3.1 (Ronquist & Huelsenbeck 2003). Metropolis-coupled Markov chains (MCMCs) with Monte Carlo simulation were run until likelihoods remained stationary and the two independent runs converged after one million generations. By sampling every 100th generations from the two independent runs in MrBayes and discarding the first 25% of the trees as burn-in, 50% majority-rule consensus phylograms were constructed. Molecular clock and species tree inference were implemented in BEAST package v.2.1 (Drummond & Rambaut 2007). First, strict, relaxed lognormal and exponential clock models were analysed for each *locus* considering a model of coalescent constant population. The Bayesian inference was made with MCMC chains of 4 x 10^7^ states (or 1 x 10^8^ states if convergence was not reached) and sampling trees every 5,000 states. Relaxed exponential and strict clocks were compared using Bayes factor (BF), which was calculated using Tracer software with 1,000 random bootstrap replications to estimate marginal likelihood. Second, a Bayesian co-estimation of the species tree and molecular clock parameters was made for the *loci* analysed by Diosque et al. (2014) using a STAR-BEAST analysis. Third, a calibration point was considered in the analysis for those *loci* whose homologous sequences were present in *Trypanosoma brucei* strain TREU427 genome and that were informative about DTU relationships. To calibrate the clock-rate estimations, a normally distributed prior of the divergence time between *T. brucei* and *T. cruzi* sequences with a mean of 100 million years ago and standard deviation of 2.0 was imposed as previously suggested (Lewis et al. 2011). Clock models were unlinked and the implemented model for each *locus* was selected according to the BF analysis for each gene fragment. The population function in multispecies coalescent parameters was set to linear with a constant root. An MCMC chain of 250 million iterations was run, with parameters and trees sampled every 5,000 iterations and removal of the first 10% of states as burn-in. Log-files were checked for sufficient effective sampling sizes using TRACER v.1.5 (Rambaut & Drummond 2007).

Because the inclusion of genotypic data of hybrid DTUs (TcV and TcVI) can lead to bias in the phylogenetic analyses, we first obtained patterns for non-hybrid lineages (TcI to TcIV) based on the MLST allelic profiles of sequences analysed by Diosque et al. (2014). Next, six hypothetical TcII/TcIII hybrid strains with heterozygous profiles were included in the analysis. A distance matrix was generated based on the number of different alleles between strains. In addition, the distance between heterozygous and homozygous genotypes at each *locus* was considered 1 if no alleles were shared and 0.5 if one allele was shared. When two heterozygous genotypes were identical, the distance was considered 0. NJ analyses using the PHYLIP package (Felsenstein 2005) were performed based on the distance matrices.

The NJ method was also implemented to evaluate phylogeny of online available *CytB* sequences. In addition, the same method was used to analyse sequences published by Westenberger et al. (2005) and an outgroup sequence. Branch support was evaluated using 1,000 bootstrap replications.

The allele sequences for TcV and TcVI strains published by Diosque et al. (2014) were inferred for each one of the 13 *loci* with the PHASE algorithm implemented in DNAsp (Librado & Rozas 2009). We analysed 10,000 iterations sampling every each 100 states and discarding the first 1,000 as burn-in.

## RESULTS AND DISCUSSION

*TcI, TcIII and TcIV form a monophyletic group* - Based on combined data analysis of previously published information, we propose that TcI, TcIII and TcIV form a monophyletic group. In addition, we will review and discuss various models describing the relationships between the TcI, TcII, TcIII and TcIV DTUs.

**Fig. 1:**
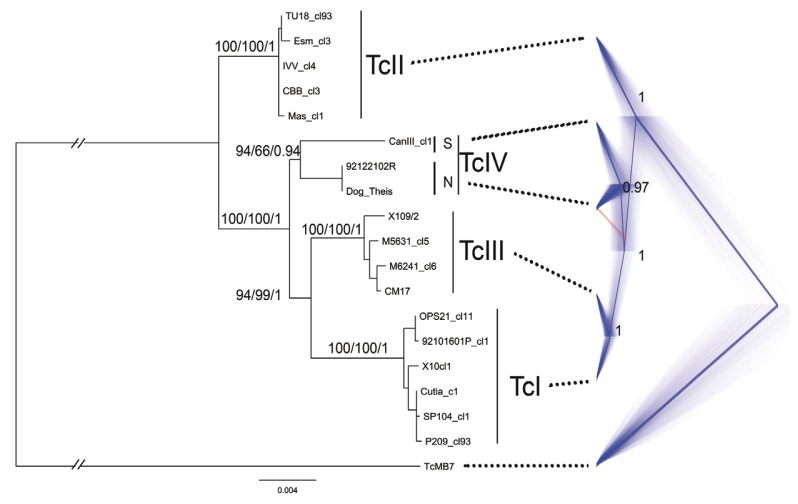
Trypanosoma cruzi phylogeny based on 13 gene fragments. Left, maximum likelihood (ML) based on concatenated sequences of 13 fragments of housekeeping genes. Branch values represent statistical support for 1,000 bootstrap repetition in a neighbour-joining analysis (1st value), 1,000 bootstrap repetitions for ML (2nd value) and posterior probability in Bayesian inference using MrBayes software (3rd value). Right, most probable topologies visualised in Densitree 2.1 to illustrate the statistical uncertainty of the species tree estimation. Greater topological agreement is visualised by a higher density of trees, whereas uncertainty in the height and distribution of nodes are represented by increased transparency. Most common topology is shown in blue and the second most common topology is shown in red. Solid blue lines represent the consensus tree and node values indicate posterior probability.

First, we analysed sequence data from 18 *T. cruzi* reference strains (Supplementary Table I, 1st 18 strains) and the *T. cruzi marinkellei *outgroup to address the phylogenetic relationships between the TcI, TcII, TcIII and DTUs. We did not include the TcV and TcVI strains because there is sufficient evidence identifying them as TcII/TcIII hybrids (Brisse et al. 1998, Sturm et al. 2003, Westenberger et al. 2005, Lewis et al. 2009, 2011). Thirteen *loci* [described in Diosque et al. (2014)] (see also the "Analysed sequences" section in Materials and Methods) were ana- lysed by different phylogenetic methods. We did not detect major incongruences between *loci *that allowed concatenation (bioNJ-ILDp = 0.855). The resulting phylogeny is shown in Fig. 1 (left tree). Two major clades were observed. The first clade clustered TcII strains, whereas the second branch clustered TcI, TcIII and TcIV DTUs. Both major branches of the tree have maximum statistical support NJ, ML and Bayesian inference (branch values on left tree in Fig. 1). The analysis for each *locus *showed that the TcI-TcIII-TcIV clade was observed in nine out of the 13 gene trees according to the ML or NJ methods (data not shown). These results provide strong evidence that TcI, TcIII and TcIV cluster in a monophyletic group.

We obtained certain topological incongruences among the trees of each *locus* (data not shown) and thus we performed a Bayesian inference of the species tree based on multilocus sequence data using a STAR-BEAST analysis. This method considers coalescent models and is an alternative method that allows us to infer the species tree, but avoid possible bias due to concatenation of sequences. The obtained species tree corroborated the observed clustering of TcI, TcIII and TcIV with high Bayesian probability (BP) (Fig. 1, right tree).

Machado and Ayala (2001) were the first to propose the TcI-TcIII-TcIV clade. They analysed sequence data of two nuclear genes (*dhfrs* and *TR*) and one maxicircle region (including the genes *COII* and *Nd1*). In this study, Machado and Ayala (2001) also observed clustering of the TcI, TcIII and TcIV DTUs based on the three analysed fragments. Although the use of just three genomic regions may not be representative of the whole genome, this was the first evidence of the TcI-TcIII-TcIV clade. Subsequently, Flores-Lopez and Machado (2011) ana- lysed the sequences of 31 nuclear *loci* and one maxicircle *locus* in seven reference strains. They analysed the tree topology for each *locus* and observed the TcI-TcIII-TcIV cluster at 24 out of the 32 *loci*. The analysis of the concatenated sequences clearly showed the same cluster with high statistical support. Although seven strains may be considered a low number of strains, these results strongly agree with what we observed.

*Unsupported models of inter-DTU relationships* - Additional models have been proposed to explain the relationships between TcI to TcIV DTUs. These models do not agree with the clustering of TcI-TcIII-TcIV.

**Fig. 2:**
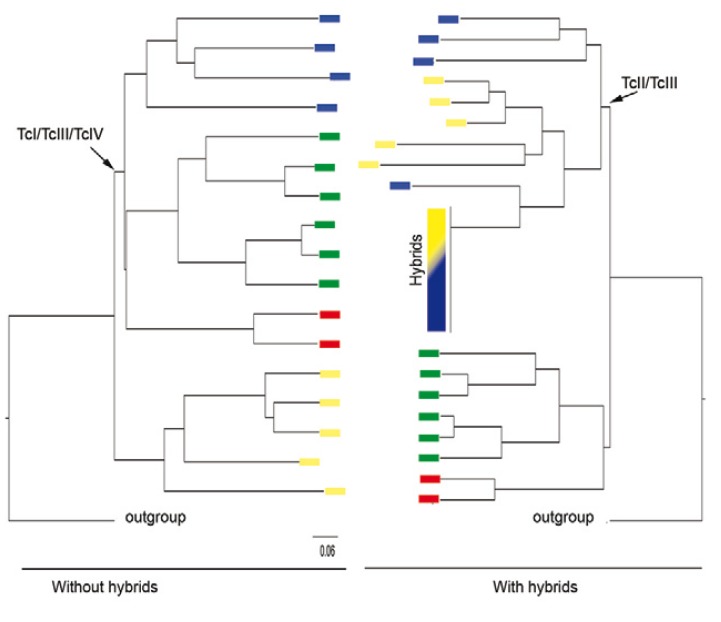
trees showing bias due to including genotypic data of hybrid strains. The left tree correspond to a neighbour-joining tree based on a simulated multilocus enzyme electrophoresis dataset. This dataset was based on multilocus sequence typing allelic profiles of 13 loci corresponding to discrete typing units TcI (green boxes), TcII (yellow boxes), TcIII (blue boxes) and TcIV (red boxes). The right tree shows a biased topology due to including of hypothetical hybrid profiles resulting of TcII and TcIII hybridisation. It can be observed that TcIII does not cluster with TcI and TcIV as in the left tree.

Brisse et al. (2000) were the first to propose a division of *T. cruzi *into six lineages. They also analysed the phylogenetic relationships among these different DTUs with MLEE and random amplified polymorphic DNA (RAPD). Specifically, they analysed 22 *loci* by MLEE and 20 different primers by RAPD. Two major lineages were observed for both markers with high bootstrap support. The first lineage corresponded to TcI and the second one corresponded to a cluster of TcII to TcVI (previously called TcIIa to TcIIe). However, a major concern about the phylogenetic analysis made by Brisse et al. (2000) is the inclusion of genotypic data from TcV and TcVI. Considering the hybrid status of TcV and TcVI, there may have been an artefact in the tree inference because genotypic data of hybrids was included in the analysis. As we do not have MLEE data available for *T. cruzi*, we conducted a simple analysis to test the hypothesis of a biased phylogenetic inference. Based on the sequences of the 13 gene fragments analysed by Diosque et al. (2014), we generated MLST allelic profiles for strains from TcI to TcIV (Supplementary Table I, strains 1-18). The NJ algorithm revealed two major clades: TcI-TcIII-TcIV and TcII (Fig. 2, left). Additionally, we included six hypothetical hybrid strains in the analysis. These "hybrid" strains have allelic profiles compatible with a hybridisation event between TcII and TcIII (i.e., TcII = allele1, TcIII = allele2 and hybrid strains = allele1/allele2). The NJ indicated two major clusters, but TcIII did not cluster with TcI. Instead, TcIII strains clustered with TcII and the hybrids (Fig. 2, right). This simple example clearly shows that genotypic data of hybrid DTUs should be cautiously considered to avoid the inference of a biased phylogeny.

**Fig. 3:**
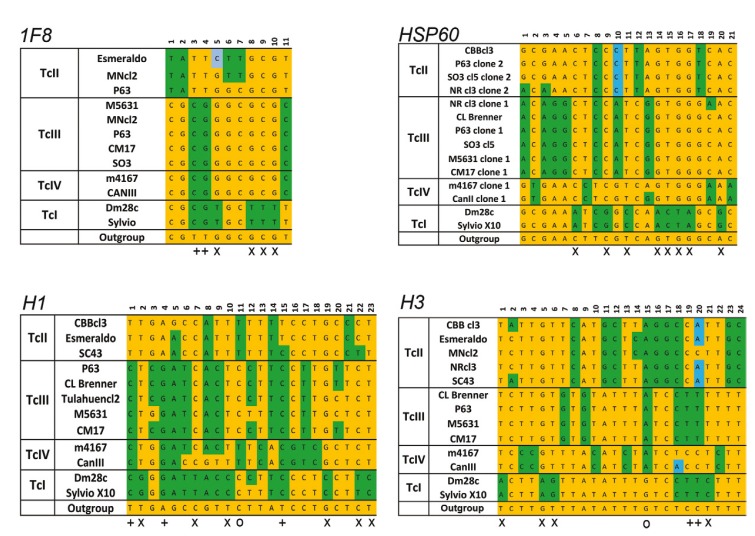
apparent mosaic patterns observed by Westenberger et al. (2005). Polymorphic sites for 1F8 calcium-binding protein, histone H1, histone H3 and heat-shock protein 60 (HSP60) loci analysed by Westenberger et al. (2005) plus the outgroup sequences. Coloured columns show polymorphic sites with information on clustering of different strains (only parsimony-informative sites are shown). Green bases represent a derived character, whereas yellow bases indicate an ancestral feature. Note that excluding the outgroup, the sites marked with an X wrongly appear to cluster TcIII and/or TcIV with TcII. In contrast, positions denoted with + show clustering of TcIII and/or TcIV with TcI according to the outgroup. Positions denoted with an "o" are clustering TcIV and/or TcIII with TcII. In consequence, excluding the outgroup gives an apparent mosaic which is not real.

Westenberger et al. (2005) proposed an alternative evolutionary framework for *T. cruzi*. This alternative model proposes that TcI and TcII are ancestral lineages and a first hybridisation event occurred between these DTUs. In addition, they proposed that the hybrid descendant underwent a genomic loss of heterozygosity and/or recombination between parental alleles. This genomic process would have formed the TcIII and TcIV DTUs. Westenberger et al. (2005) presented evidence supporting this model. In four out of nine gene sequences they observed that the genetic distance from TcIII and/or TcIV to TcII was shorter than that to TcI. In fact, five *loci* showed the inverse pattern. In addition, they proposed that TcIII and TcIV have mosaic patterns combining different fragments of TcI and TcII sequences. In their analyses, Westenberger et al. (2005) did not include an outgroup. In the absence of an outgroup it is not possible to determine whether a character is derived or ancestral. Unfortunately, relationships among DTUs cannot be clearly addressed under this scenario of uncertain ancestry. To clarify the relationships between the TcI to TcIV DTUs we reanalysed several *loci* examined by Westenberger et al. (2005), particularly those that were proposed to provide evidence of clustering of TcII with TcIII and/or TcIV. In addition, we included an outgroup sequence corresponding to *T. cruzi marinkellei* for each *locus*. Finally, we also evaluated the presence or absence of mosaic patterns. Apparent mosaic patterns were observed before including the outgroup sequence (Fig. 3, sites denoted with an x-mark). However, we did not observe any mosaic for any *locus* when the outgroup was included in the alignment (Fig. 3). Seven informative sites (denoted with a plus sign in Fig. 3) favoured the clustering of TcIII and/or TcIV with TcI. Instead, just one polymorphism clustered TcII with TcIV and one polymorphism clustered TcII-TcIII-TcIV. These two last sites were located at different *loci*; thus, homoplasy is the most parsimonious explanation for their existence. We also analysed phylogenetic trees for these four *loci*. *H1* and *1f8* genes showed clear clustering of TcI-TcIII-TcIV with strong support (Supplementary Figure). In contrast, *H3* and *HSP60* showed clusters that were incompatible with TcI-TcIII-TcIV. However, these clusters showed low bootstrap support (< 70%), suggesting a low phylogenetic signal to address inter-DTU relationships (Supplementary Figure).

**Fig. 4:**
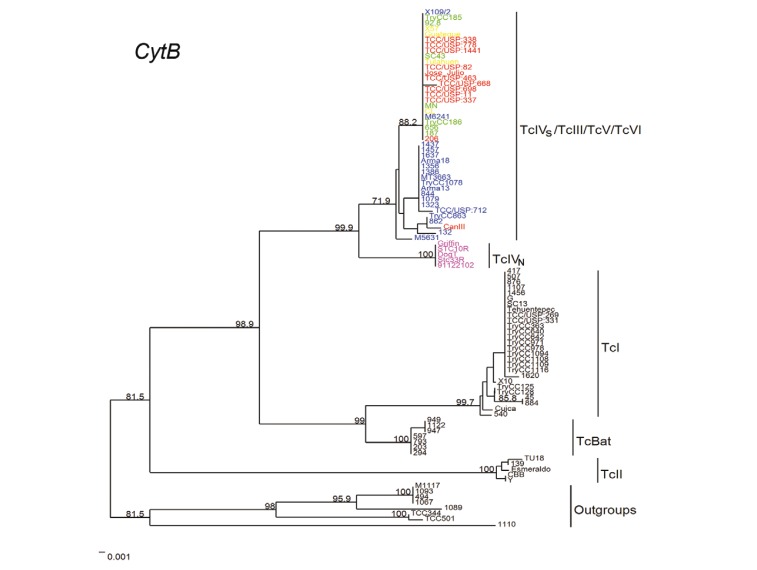
neighbour-joining tree based on cytochrome B (CytB) sequence of 97 different strains downloaded from GenBank. Branch values represent bootstrap percentage over 1,000 replications. Different discrete typing units (DTUs) are indicated with vertical bars (TcIVS: strains TcIV from South America; TcIVN: strains TcIV from North America). Some sequence names were coloured to show the corresponding DTU (blue: TcIII; red: TcIVS; violet: TcIVN; green: TcV; yellow: TcVI).

Consequently, this reanalysis of Westenberger's et al. (2005) data including an outgroup revealed that the analysed TcIII and TcIV sequences have no mosaic patterns. In addition, this reanalysis supports the clustering of TcIII and TcIV with TcI. These results highlight the usefulness of using one or more outgroup strains in phylogenetic analyses of *T. cruzi* strains.

de Freitas et al. (2006) proposed the three ancestor model for the evolution of *T. cruzi*. They analysed several strains of TcI, TcII, TcIII, TcV and TcVI. However, few strains of TcIV were analysed and this DTU was not considered in the model. Sequences from three maxicircle *loci* (*COII*, *Nd1* and *Cyt B*) and five microsatellite *loci* were analysed. They proposed the existence of at least three ancestral lineages (TcI, TcII andTcIII). However, no outgroup was included in this study and thus they could not define the relationships among these three ancestors. Machado and Ayala (2001) showed that for the *COII-Nd1* *locus* [which was also analysed by de Freitas et al. (2006)], the TcI-TcIII-TcIV cluster is clearly observed. Consequently, we also analysed 97 *CytB* sequences that are available in GenBank and included several TcIV strains and outgroup sequences corresponding to *T. cruzi marinkellei* and *Trypanosoma vespertilionis*. We also observed that *cytB* phylogeny strongly supported the clustering of the TcI, TcIII and TcIV DTUs (bootstrap = 98.9) (Fig. 4). Consequently, mitochondrial* loci* analysed by de Freitas et al. (2006) also support the TcI-TcIII-TcIV cluster.

*TcI and TcIII are sister clades* - We collected evidence from nuclear genome data showing that TcI and TcIII share a common ancestor. First, there was strong support for this cluster (NJ bootstrap = 94, ML bootstrap = 99 and BP = 1) according to the 13 *loci* phylogeny observed in Fig. 1 (left). Topologies showing the TcI-TcIII cluster were the most frequently resolved type among the 13 *loci* analysed (data not shown). Six and four *loci *showed TcI-TcIII clusters for individual gene trees inferred by NJ and ML, respectively (data not shown). In contrast, four (NJ) and three (ML) individual gene trees were incompatible with this cluster (data not shown). The remaining topologies (3 for NJ and 6 for ML analysis) were unresolved about the TcI-TcIII-TcIV relationships. The low number of *loci *indicating clustering of TcI-TcIII suggests that both lineages rapidly diverged after the TcI-TcIII ancestor was separated from that of TcIV. The species tree obtained by Bayesian inference also strongly supported this clustering (Fig. 1, right). However, the TcIII-TcIV cluster observed for few *loci* may suggest incomplete lineage sorting, but additional data are required to confirm this hypothesis. Homoplasy and lateral gene transfer are alternative hypotheses.

Additional evidence of the TcI-TcIII clustering was provided by Machado and Ayala (2001). They observed that the *dhfrs* and *TR* *loci* clustered both DTUs together. In addition, the same pattern was observed for the *GPI* *locus* (Lewis et al. 2011). Flores-Lopez and Machado (2011) observed the TcI-TcIII cluster on the phylogeny of 32 concatenated *loci* (bootstrap = 72, Bayesian support = 100). In addition, 11 out of 24 topologies that supported TcI-TcIII-TcIV clustering also supported clustering of TcI-TcIII. Just six topologies were incompatible with the TcI-TcIII clustering (3 showed TcI-TcIV clustering and 3 showed TcIII-TcIV clustering). Finally, the *H1* and *H3* *loci* shown in Supplementary Figure also support the clustering of TcI and TcIII.

*TcIV is divided into two main sub-clusters: TcIV* *_S_* *and TcIV* *_N_* - Fig. 1 shows considerable distance between the CanIII strain (from Brazil, TcIV_S_) and TcIV strains from North America (TcIV_N_). Eleven out of the 13 ana- lysed *loci* clustered the TcIV_N_ strains separately from the TcIV_S_ strain. In addition, the *cytB* analysis (Fig. 4) showed that TcIV_N _was clearly separated from TcIV_S_ sequences, which was also observed by others (Brisse et al. 2003, Marcili et al. 2009a, b, Ramirez et al. 2011). Evidence for this split was previously described by different makers: MLEE and RAPD (Brisse et al. 2000), rDNA promoter region (Brisse et al. 2003), SSU rDNA (Marcili et al. 2009b), *Dhfrs* sequence (Roellig et al. 2013), GPI sequence (Lewis et al. 2011) and multilocus analyses (Yeo et al. 2011, Messenger et al. 2012).

*Multiple introgression events from TcIV* *_S_* *to TcIII explain the TcIII kDNA origin* - As we proposed, TcI and TcIII form a monophyletic group according to the nuclear phylogeny. However, mitochondrial data showed clustering of TcIII with TcIV_S_ through an analysis of the *COII-Nd1 locus* (Machado & Ayala 2001, Lewis et al. 2011), *cytB* (Marcili et al. 2009a, b) and MLST of kDNA (kMLST) (Messenger et al. 2012). These results support a mitochondrial introgression of TcIV_S_ into the TcIII lineage. There are several pieces of evidence indicating that mitochondrial introgression currently occurs in *T. cruzi* and DTU TcIV may be the kinetoplast donor. Messenger et al. (2012) reported two strains that closely clustered with certain TcI strains according to 25 microsatellite *loci*, but they clustered with TcIV_S_ according to kMLST. These authors proposed a recent event of mitochondrial introgression of TcIV_S_ into the TcI genome. In addition, Roellig et al. (2013) observed eight events of introgression in North American *T. cruzi* isolates. In these cases, strains with a TcI nuclear genotype clustered with TcIV_N_ according to the analysis of the *COII-Nd1* kDNA fragment. The same pattern was observed for an isolate from Bolivia (GPI genotype = TcI, Nd1 genotype = TcIV_S_) (Barnabe & Breniere 2012). These results suggest that mitochondrial introgression is not an exceptional phenomenon in *T. cruzi* and it appears occur more frequently from TcIV to other lineages.

**Fig. 5:**
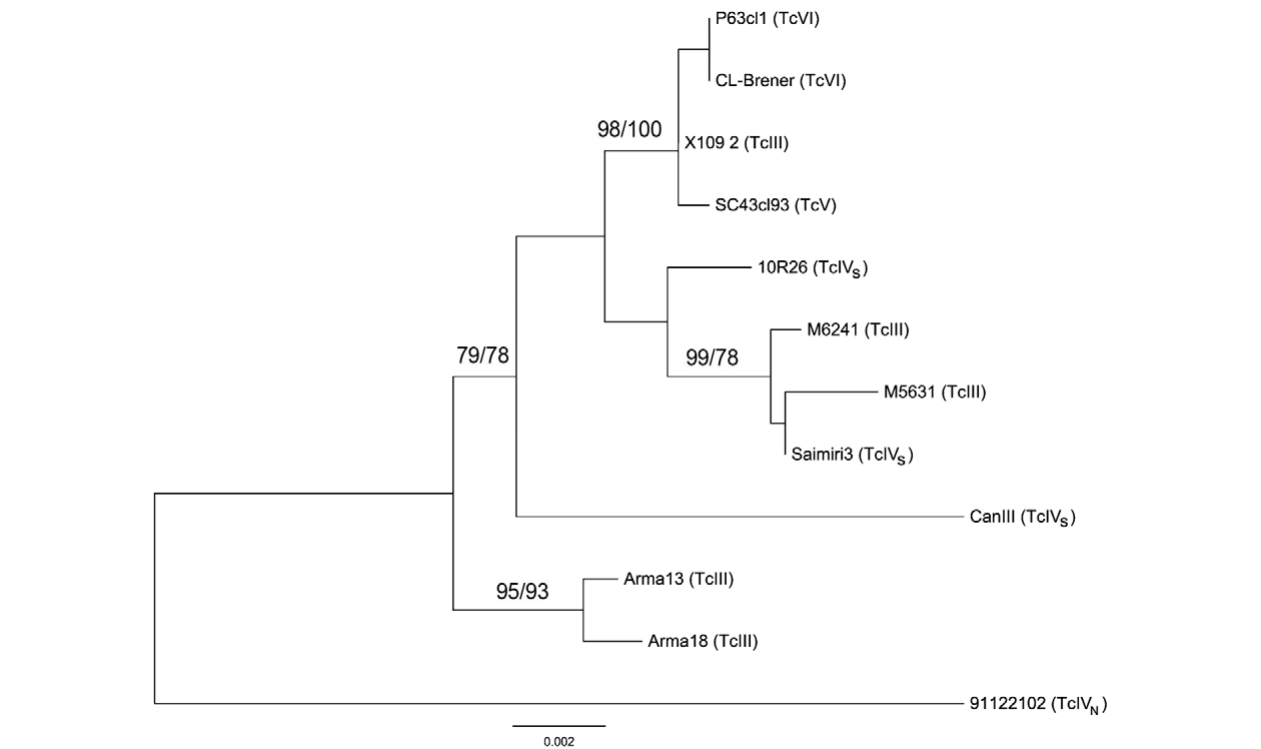
maximum likelihood (ML) tree based on concatenation of cytochrome B (CytB) and the cytochrome c oxidase subunit II-NADH dehydrogenase 1 kDNA fragments for TcIII and TcIV strains. The same topology was obtained by neighbour-joining (NJ) method. The corresponding lineage for each strain is shown between brackets. Branch values indicates 1,000 bootstrap replications for NJ (1st value) and ML (2nd value).

Based on the *COII-Nd1* sequence, Lewis et al. (2011) proposed that multiple introgression events might have occurred between TcIII and TcIV_S_. Here, we collected evidence supporting the occurrence of multiple events of introgression in the evolutionary history of TcIII. If only one introgression event occurred into an ancestral TcIII, TcIII strains should be clustered together in a sister clade to TcIV_S_ when kinetoplast sequences are analysed. However, we observed at least two clusters grouping TcIII and TcIV strains in analysis of the *cytB* *locus *(Fig. 4). Consequently, we analysed a set of 11 strains corresponding to the TcIII, TcIV_S_, TcV and TcVI DTUs (Supplementary Table II) for three mitochondrial *loci* (*Nd1*, *COII* and *CytB*) with available sequences. We also included a TcIV_N_ sequence as an outgroup. We observed that the TcIII-TcV-TcVI strains did not cluster into a single branch (Fig. 5). Instead, the TcIII-TcV-TcVI strains clustered into three different and strongly supported branches (Fig. 5). This observation may not be explained by a single introgression event and thus must have been caused by several.

There are a few explanations for the observed incongruence among nuclear and mitochondrial phylogenies. Incomplete lineage sorting is an unlikely explanation. Under incomplete lineage sorting hypothesis, because genetic exchange should have been at least of moderate frequency for the TcI/TcIII/TcIV ancestor. In addition, kDNA should have diverged into three sequence groups (TcI, TcIV_S_-TcIII and TcIV_N_) before the separation of TcI-TcIII and TcIV. This hypothesis accounts for the observed nuclear-mitochondrial incongruence. However, under the incomplete lineage sorting hypothesis, a large distance between TcIII and TcIV strains is expected because kDNA diverged before the separation of the TcI-TcIII-TcIV cluster. Instead, the genetic distances between some TcIII and TcIV strains (Fig. 5) are relatively short (i.e., just one differential SNP is observed between M6241-TcIII and Saimiri3-TcIV_S_). Another hypothesis is that hybridisation events between TcIII and TcIV_S_ were followed by several backcrosses of the hybrid strain with TcIII strains. In addition, because all TcIII strains analysed have a different TcIV_S_ kDNA, it is expected that introgression occurred during TcIII lineage expansion and not just at the origin of the lineage. It is also likely that TcIV was already widely distributed before TcIII expansion (Fig. 1, Supplementary Table III). This scenario of mitochondrial introgression during a species expansion was theoretically analysed few years ago. Currat et al. (2008) proposed a demographic neutral model that predicts that when one species invades an area already occupied by a related species, asymmetrical introgression may occur mainly from the local species towards the invader. Asymmetrical mitochondrial introgression was observed for several animal and plant species (Currat et al. 2008) and even in algae (Neiva et al. 2010). In addition, the model also predicts that introgression should be more frequent for DNA fragments with lower intra-species gene flow. In this sense, mitochondrial introgression is more probable than nuclear introgression in organisms with uniparental inheritance of mtDNA because of the lower gene flow among populations for the mitochondrial genome (Du et al. 2011). kDNA is of uniparental inheritance in *T. cruzi* hybrids, hence the kDNA should have lower interpopulation gene flow than a biparentally inherited *locus *if genetic exchange was of at least moderate frequency. Consequently, if the genetic exchange had a moderate frequency at least at the expansion front of TcIII, the model proposed by Currat et al. (2008) may perfectly explain the multiple asymmetrical mitochondrial introgression events observed for TcIII. Although true sexual mechanisms (meiosis-dependent) have not yet been described for *T. cruzi* and preponderant clonality is widely accepted (Tibayrenc & Ayala 2013), population data suggest that frequent genetic exchange may occur in certain restrained populations (Ocana-Mayorga et al. 2010, Baptista et al. 2014). Alternatively, an unconventional mechanism of mitochondrial transfer may explain kDNA transfer, although no such mechanism has been described for any organism thus far. Whatever the mechanism of introgression, if TcIII was at expansion, the allele surfing hypothesis (Klopfstein et al. 2006) (called here kDNA surfing) may be a good explanation for fixing the introgression. The surfing hypothesis proposes that a rare allele originated on the edge of a wave of expansion may be propagated by the wave reaching high frequencies or even fixation far away from its origin. In this sense, the introgressed kDNA may have been propagated by the wave of expansion and lead to it being fixed for the whole TcIII DTU. The multiple introgressions observed for TcIII may be explained by this model and positive selection may not be invoked (although it may be implicated).

Any introgression hypothesis requires at least some overlap between the ecological niches of both DTUs. Although different ecological niches have been proposed for TcIV_S_ (arboreal ecotope) (Marcili et al. 2009b, c) and TcIII (terrestrial ecotope) (Llewellyn et al. 2009, Marcili et al. 2009b), an overlap of these niches is possible. In fact, *Pastrongylus geniculatus* (the main vector of TcIII in terrestrial mammals) has been reported into the arboreal ecotope in the Amazonia and even infected by TcIV (Marcili et al. 2009b). In addition, TcIV specimens have been documented to infect nine-banded armadillos (*Dasypus novemcinctus*) at least in North America (Yeo et al. 2005, Roellig et al. 2013).

An alternative to the kDNA transfer from TcIV_S_ to TcIII is introgression occurring in the opposite direction (from TcIII to TcIV_S_). For this hypothesis to be plausible, TcI kDNA must have diverged before the separation of TcIV from the TcI-TcIII-TcIV ancestor (incomplete lineage sorting) and subsequently, multiple introgressions must have occurred from TcIII to TcIV_S_. However, the most recent common ancestor (MRCA) for TcIII-TcIV kDNA should have occurred before the divergence of TcI-TcIII-TcIV. Considering the relatively short distance between TcIII-TcIV_S_ to TcIV_N_ in relation to inter-DTU relationships (Fig. 4), it is unlikely that the kDNA of both groups coalesced previous to the TcI-TcIII-TcIV divergence. Consequently, directional transfer from TcIV_S_ to TcIII is more likely.

Finally, if TcIV_S_ transferred its kDNA to TcIII, this last lineage transferred the TcIV_S_ kDNA to the hybrid DTUs TcV and TcVI.

**Fig. 6:**
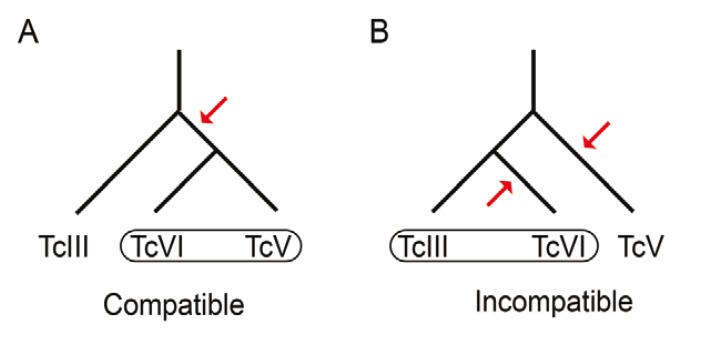
examples of haplotype topologies which are compatible with a single hybridisation event between TcII and TcIII (A) and incompatible with the hypothesis of a single hybridisation event (B). Arrows indicate when hybridisation events could have occurred in the haplotype history. Note that for A the TcV and TcVI haplotypes diverged after hybridisation event whereas in B the haplotypes diverged before hybridisation events. It is important to consider that the topology A is also compatible with multiple hybridisation events (particularly when the sampled TcIII strain is distantly related to the parental TcIII strain involved into the hybridisation). The same example applies for TcII-TcV-TcVI haplotype history.

*TcV and TcVI are hybrids originated from independent hybridisations events between TcII and TcIII* - Westenberger et al. (2005) proposed a single hybridisation event for the origin of the TcV and TcVI DTUs. Their model proposes that after the hybridisation event between TcII and TcIII, the hybrid lineage diverged into the current DTUs TcV and TcVI. This was the most likely hypothesis according to their data. However, several data suggest that two independent hybridisations occurred between TcII and TcIII. de Freitas et al. (2006) were the first to propose that two independent hybridisation events gave rise to TcV and TcVI, based on the extensive differences between TcV and TcVI haplotypes. In addition, if the hypothesis of a single hybridisation event were correct, TcV and TcVI would be expected to cluster together in a branch (Fig. 6A). Instead, the occurrence of at least two hybridisation events is supported by the clustering of one hybrid with its parental for any allele (Fig. 6B). Machado and Ayala (2001) analysed the *COII-Nd1* fragment sequence and observed for DTU TcVI that TcIII-like alleles clustered with TcIII strains instead of TcV (the same pattern exemplified in Fig. 6B). In addition, we analysed haploid sequences (inferred by PHASE) of 16 reference strains from the TcII, TcIII, TcV and TcVI DTUs (Supplementary Table I, strains 7-15 and 19-25). The TcV-TcVI cluster was observed only for two *loci* (*Rb19* and *Rho1*), whereas clustering incompatible with the TcV-TcVI group was observed in six *loci*. Incompatibilities in one out of these six *loci* may be attributed to intralocus recombination in TcV (*Rb19*). However, the remaining five loci (*CoAR*, *Met-II*, *MPX*, *Sod-B* and *Sttpf-2*) clearly showed topologies similar to Fig. 6B, which provides evidence against a single hybridisation event (data not shown). These results are in agreement with the work of Flores-Lopez and Machado (2011). They showed that TcV and TcVI do not form a monophyletic group for TcII-like alleles (TcV clustered with TcII; branch support: bootstrap = 90, BP = 1). We reviewed individual topologies for 30 *loci* analysed by Flores-Lopez and Machado (2011) and observed that 50% were incongruent with the clustering of TcV and TcVI. Instead, just four topologies grouped TcV and TcVI in a monophyletic branch. Finally, Lewis et al. (2011) observed for 28 microsatellite *loci* that most of microsatellite alleles that discriminated between TcV and TcVI were also present in parental DTUs. If those alleles originated by divergence after the hypothetical TcV/TcVI ancestor, the occurrence of the same alleles in parental strains would require several homoplasy events which is a less parsimonious hypothesis. Consequently, the hypothesis of independent events is more parsimonious than the hypothesis of repeated homoplasy.

*About phylogenetic position of TcBat* - Recently, a bat-associated lineage has been described based on *cytB* and a few nuclear genes (Marcili et al. 2009a, Pinto et al. 2012). This lineage was proposed to be closely related to TcI (Marcili et al. 2009a, Pinto et al. 2012). In this sense, additional markers such as nuclear MLST and kMLST will help to confirm this phylogenetic position of TcBat. Interestingly, Guhl et al. (2014) proposed that this group is an ancestor for all DTUs, based on four kDNA fragments and four nuclear *loci*. They only showed a phylogenetic tree of *CytB* showing this basal position. In contrast, we observed that TcBat does not have a basal position based on an analysis of *CytB* (Fig. 4) and our observation is in agreement with results of Marcili et al. (2009a) and Pinto et al. (2012). In addition, branch lengths and branch support were not reported by Guhl et al. (2014) to support the accuracy of the phylogenetic inference. This conclusion may be biased due to an incorrect selection of the model used in Bayesian inference. They implemented a strict molecular clock for the *cytB* *loci* although the p value reported by them (using the likelihood ratio test) rejected it. Unfortunately, no sequence for any *loci* was uploaded to GenBank and we could not repeat their analyses.

*Estimating dates for T. cruzi evolutionary history* - The first paper dating the age of *T. cruzi* proposed an ancient origin for the parasite (Briones et al. 1999). The MRCA for *T. cruzi* and *T. cruzi marinkellei* was dated at approximately 200-475 million years ago and the MRCA of the *T. cruzi* lineages was dated at 33-88 million years ago. However, most recent papers questioned the ancient origin hypothesis and proposed that the origin was very recent (Flores-Lopez & Machado 2011, Lewis et al. 2011).

**Fig. 7:**
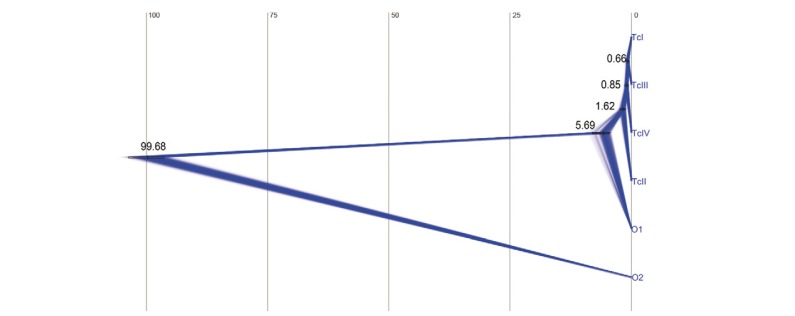
Trypanosoma cruzi species tree showing divergence dates. Most probable topologies were visualised in Densitree 2.1 to illustrate the statistical uncertainty of the species tree inference and date estimation. Time of the most recent common ancestor is shown above nodes. Horizontal bars represent 95% high posterior density. Vertical bars divide the tree every 25 million years. O1: T. cruzi marinkellei; O2: T. brucei strain TREU927.

We estimated divergence times for the phylogeny of *T. cruzi* by analysing nine out of the 13 MLST *loci* using BEAST software. A relaxed clock was favoured for eight/nine *loci* according the BF (> 0.5) (Supplementary Table III). Divergence times were considerably higher (Supplementary Table III) than was recently reported for different splits observed in the phylogenetic tree of *T. cruzi* (Flores-Lopez & Machado 2011, Lewis et al. 2011). However, divergence times had high confidence intervals, which reveal high uncertainty for age estimation. The high intervals may be due to the low information level for each single *locus.* Consequently, we performed a STAR-BEAST analysis to combine information on different *loci* and make a joint estimation of the species tree and divergence dates. A similar topology to Fig. 1 was observed for inter-DTU relationships and we confirmed monophyly for clusters TcI-TcIII-TcIV and TcI-TcIII. Divergence times for inter-DTU relationships are shown in Fig. 7.

**Fig. 8:**
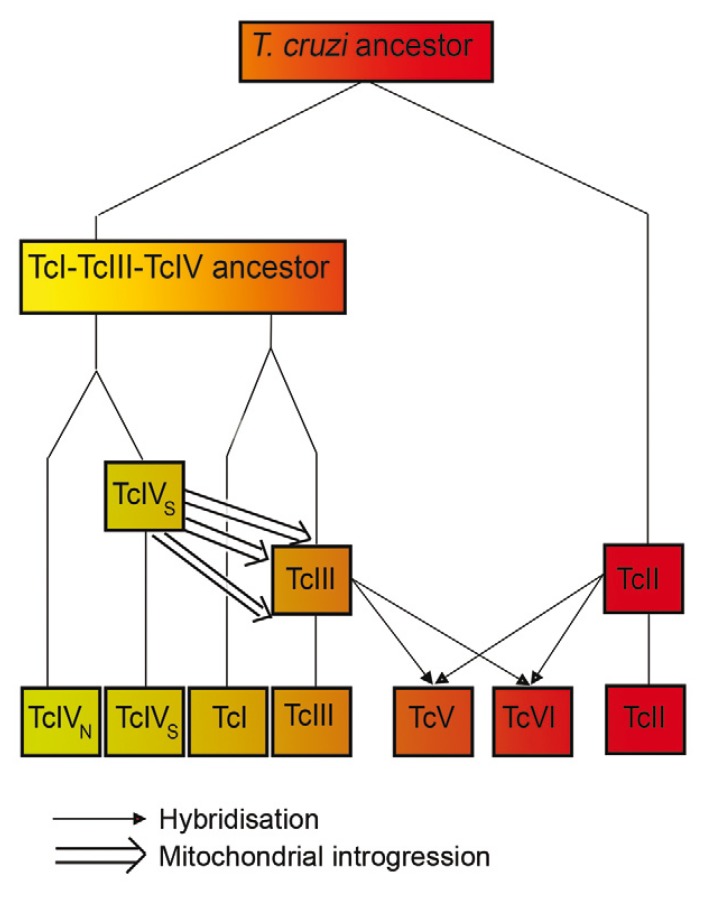
the revisited model for Trypanosoma cruzi evolution.

*The T. cruzi evolution model* - The proposed model is shown in Fig. 8. According to our analyses, the *T. cruzi *ancestor was separated from *T. cruzi marinkellei* approximately five-seven million years ago . This ancestor diversified approximately one-three million years ago into two different groups: TcII and TcI-TcIII-TcIV. TcIV separated first from the latter clade and, after this separation, TcIV diverged into two geographically differentiated groups (TcIV_S_ and TcIV_N_). Subsequently, TcI- TcIII was divided into two different clades (0.37-1 million years ago). Incomplete lineage sorting may explain the existence of some topologies clustering TcIII and TcIV, although additional genes should be analysed to confirm this. After the TcI-TcIII split, TcIV_S_ transferred the kinetoplast to TcIII by an unknown mechanism of mitochondrial introgression. According to the proposed model, multiple introgression events occurred after the split of TcI-TcIII clade and the TcIV_S_ kDNA surfed on the expansion wave of TcIII, which became fixed in the modern TcIII. In addition, the model of asymmetrical introgression for a range-expanding population may fit well to the observed kDNA pattern, although further data should be collected to test this hypothesis. Finally and most recently, two independent hybridisation events between TcII and TcIII gave origin to the TcV and TcVI DTUs. Both of them are carriers of TcIV_S _kDNA.
